# Hepatocellular carcinoma: Advances in systemic therapies

**DOI:** 10.12688/f1000research.145493.1

**Published:** 2024-02-16

**Authors:** Trevor Kwan-Hung Wu, Rex Wan-Hin Hui, Lung-Yi Mak, James Fung, Wai-Kay Seto, Man-Fung Yuen

**Affiliations:** 1 Department of Medicine, School of Clinical Medicine, The University of Hong Kong, Hong Kong, Hong Kong; 2 State Key Laboratory of Liver Research, The University of Hong Kong, Hong Kong, Hong Kong; 3 Department of Medicine, The University of Hong Kong-Shenzhen Hospital, Shenzhen, China

**Keywords:** HCC, Systemic therapy, TKI, ICI, Liver, Neoadjuvant, Adjuvant

## Abstract

Advanced hepatocellular carcinoma (HCC) is traditionally associated with limited treatment options and a poor prognosis. Sorafenib, a multiple tyrosine kinase inhibitor, was introduced in 2007 as a first-in-class systemic agent for advanced HCC. After sorafenib, a range of targeted therapies and immunotherapies have demonstrated survival benefits in the past 5 years, revolutionizing the treatment landscape of advanced HCC. More recently, evidence of novel combinations of systemic agents with distinct mechanisms has emerged. In particular, combination trials on atezolizumab plus bevacizumab and durvalumab plus tremelimumab have shown encouraging efficacy. Hence, international societies have revamped their guidelines to incorporate new recommendations for these novel systemic agents. Aside from treatment in advanced HCC, the indications for systemic therapy are expanding. For example, the combination of systemic therapeutics with locoregional therapy (trans-arterial chemoembolization or stereotactic body radiation therapy) has demonstrated promising early results in downstaging HCC. Recent trials have also explored the role of systemic therapy as neoadjuvant treatment for borderline-resectable HCC or as adjuvant treatment to reduce recurrence risk after curative resection. Despite encouraging results from clinical trials, the real-world efficacy of systemic agents in specific patient subgroups (such as patients with advanced cirrhosis, high bleeding risk, renal impairment, or cardiometabolic diseases) remains uncertain. The effect of liver disease etiology on systemic treatment efficacy warrants further research. With an increased understanding of the pathophysiological pathways and accumulation of clinical data, personalized treatment decisions will be possible, and the field of systemic treatment for HCC will continue to evolve.

## Introduction

Primary liver cancer ranks as the seventh most common cancer and the third most common cause of cancer-related mortality globally. ^1^ Hepatocellular carcinoma (HCC) is the most common type of primary liver cancer, frequently occurring as a complication of chronic liver diseases, in particular, for those with established cirrhosis. Common risk factors for HCC include chronic infection by hepatitis B (HBV) or hepatitis C (HCV), alcohol-related liver disease, and steatotic liver disease. ^2^ HCC screening and surveillance, usually via ultrasound in combination with alpha-fetoprotein (AFP), aims to detect HCC at an early stage, and is associated with a higher probability of curative therapy and improved outcomes. ^3^ Resection, ablation and liver transplant remain as the curative treatment options and are associated with the best outcomes in HCC. ^4^ On the other hand, the experience in loco-regional therapies (trans-arterial chemoembolization [TACE] and stereotactic body radiation therapy [SBRT]) are continuously accumulating and treatment indications are expanding. ^5^

Despite screening efforts, many cases of HCC are still diagnosed at an advanced stage. Concomitant cirrhosis and field cancerization may preclude these patients from receiving curative or loco-regional therapies, and systemic therapy may be the only remaining treatment option. ^6^ Traditionally, pharmacological treatment for advanced HCC has been unsuccessful, primarily driven by the intrinsic resistance of HCC to conventional chemotherapy. ^7^ Nonetheless, the introduction of sorafenib in 2007 has completely revolutionized systemic therapy for advanced HCC, ^8^ and multiple clinical trials on new-generation targeted therapy and immunotherapy have been conducted ( Table 1). In response to these advancements, professional societies have revamped their guidelines on the treatment of advanced HCC ( Table 2). ^4^^,^^9^^,^^10^ This review discusses the advancements in systemic therapies for HCC, with a particular focus on targeted therapy and immunotherapy. A literature search was performed on Pubmed with the keywords “hepatocellular carcinoma,” “systemic therapy,” “targeted therapy,” “immunotherapy,” “neoadjuvant” and “adjuvant.” Papers up to November 1, 2023, were screened and included if relevant.

**Table 1.** Major trials on systemic therapy for advanced hepatocellular carcinoma.

Trial name	Years	Study drug	Control	No. of patients	Median OS in intervention group vs control (months)
Targeted therapies					
SHARP ^8^	2008	Sorafenib	Placebo	602	10.7 vs 7.9 HR 0.69, 95% CI 0.55-0.87, p<0.001
Asia-Pacific ^18^	2009	Sorafenib	Placebo	271	6.5 vs 4.2 HR 0.68, 95% CI 0.50-0.93, p=0.014
RESOURCE ^30^	2017	Regorafenib	Placebo	573	10.6 vs 7.8 HR 0.63, 95% CI 0.50-0.79, p<0.001
REFLECT ^28^	2018	Lenvatinib	Sorafenib	954	13.6 vs 12.3 HR 0.92, 95% CI 0.79-1.06, non-inferiority demonstrated
CELESTIAL ^32^	2018	Cabozantinib	Placebo	707	10.2 vs 8.0 HR 0.76, 95% CI 0.63-0.92, p=0.005
REACH-2 ^37^	2019	Ramucirumab	Placebo	292	8.5 vs 7.3 HR 0.71, 95% CI 0.53-0.95, p=0.020
Immunotherapies					
KEYNOTE-240 ^55^	2020	Pembrolizumab	Placebo	413	13.9 vs 10.6 HR 0.78, 95% CI 0.61-1.00, p=0.024
CheckMate-459 ^54^	2022	Nivolumab	Sorafenib	743	15.2 vs 13.4 HR 0.85, 95% CI 0.72-1.02, p=0.075
KEYNOTE-394 ^56^	2022	Pembrolizumab	Placebo	453	14.6 vs 13.0 HR 0.79, 95% CI 0.63-0.99, p=0.018
Combination therapies					
IMbrave150 ^64^	2020	Atezolizumab + bevacizumab	Sorafenib	501	19.2 vs 13.2 HR 0.58, 95% CI 0.42-0.79, p<0.001
ORIENT-32 ^68^	2021	Sintilimab + bevacizumab	Sorafenib	595	Median not reached at analysis vs 10.4 HR 0.57 95% CI 0.43-0.75, p<0.001
HIMALAYA ^59^	2022	Durvalumab + tremelimumab	Sorafenib	1171	16.43 vs 13.77 HR 0.78, 95% CI 0.65-0.93, p=0.004
LEAP-002 ^67^	2022	Lenvatinib + pembrolizumab	Lenvatinib + placebo	794	21.2 vs 19.0 HR 0.84, 95% CI 0.71-1.00, p=0.023
COSMIC-312 ^70^	2022	Cabozantinib + atezolizumab	Sorafenib	837	15.4 vs 15.5 HR 0.90, 95% CI 0.69-1.18, p=0.440
CARES-310 ^69^	2023	Camrelizumab + rivoceranib	Sorafenib	543	22.1 vs 15.2 HR 0.62, 95% CI 0.49-0.80, p<0.001

**Table 2.** Treatment recommendations in major guidelines.

Guideline	Key recommendations on systemic treatment	
	First line treatment	Second line treatment
EASL ^10^ (2021)	- Atezolizumab + bevacizumab - Contraindicated to atezolizumab + bevacizumab • Sorafenib • Lenvatinib	- Failed atezolizumab + bevacizumab • Multi-TKI and VEGFR2 inhibitor as per off-label availability - Failed sorafenib or lenvatinib treatment • Cabozantinib • Regorafenib • Ramucirumab (In patients with AFP ≥400 ng/mL)
BCLC ^4^ (2022)	- Atezolizumab + bevacizumab - Tremelimumab + durvalumab - Contraindicated to above two options • Sorafenib • Lenvatinib • Durvalumab	- Failed sorafenib treatment • Cabozantinib • Regorafenib • Ramucirumab (In patients with AFP ≥400 ng/mL) - Failed other first-line treatment • Clinical trials
AASLD ^9^ (2023)	- Atezolizumab + bevacizumab - Tremelimumab + durvalumab - Contraindicated to above two options • Sorafenib • Lenvatinib	- Failed atezolizumab + bevacizumab • Clinical trials • Sorafenib • Lenvatinib • Cabozantinib • Regorafenib • Nivolumab + ipilimumab - Failed tremelimumab + durvalumab • Clinical trials • Sorafenib • Lenvatinib - Failed sorafenib or lenvatinib treatment • Cabozantinib • Regorafenib • Ramucirumab (In patients with AFP ≥400 ng/mL) • Pembrolizumab • Nivolumab + ipilimumab

## Targeted therapies

### Therapeutic targets

**Figure 1.**
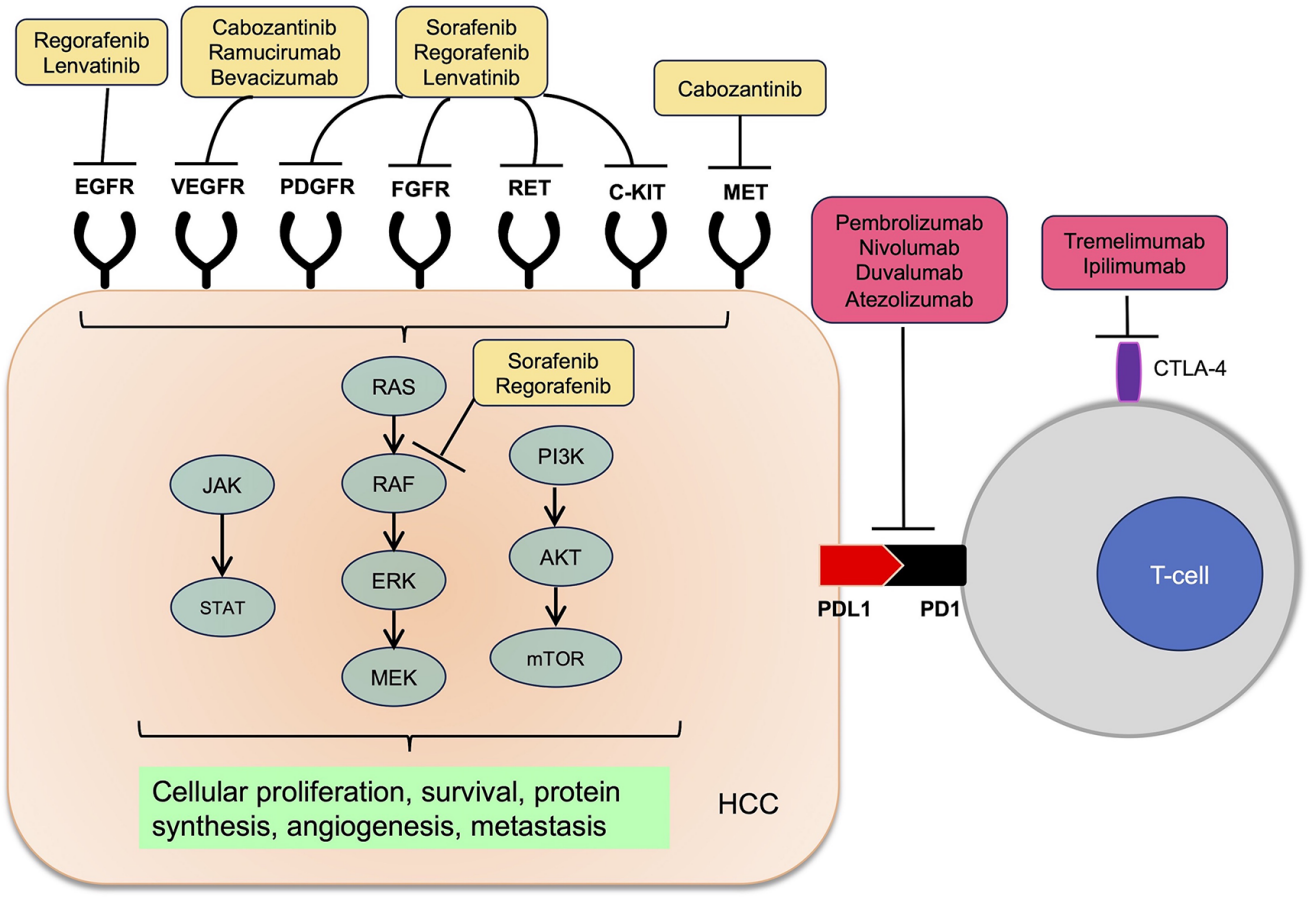
Molecular targets of systemic therapy in hepatocellular carcinoma.

Hepatocarcinogenesis is a complex multistep process involving interactions between intrinsic genetic mutations and extrinsic influence of carcinogens, which in turn drive cell cycle dysregulation, uncontrolled cellular proliferation, neovascularization, and tissue invasion. ^11^ Key pathophysiological pathways such as vascular endothelial growth factor receptor (VEGFR) signaling, RAS/RAF/MAPK pathways and TERT promoter have been identified. ^12^^–^^15^ Among the studied pathways, protein kinases serve as important molecular mediators for oncogenes, and protein kinase inhibitors have in turn become attractive therapeutic targets for HCC ( Figure 1). ^16^

### Sorafenib

Sorafenib is a tyrosine kinase inhibitor (TKI) targeting the RAS/RAF, VEGFR, and platelet-derived growth factor receptor (PDGFR) signaling pathways, thereby suppressing tumor proliferation and angiogenesis. ^17^ It was approved by the Food and Drug Administration (FDA) of the United States in 2007. Two landmark trials have validated the efficacy of sorafenib in advanced HCC. The SHARP trial is a phase III, double-blinded, placebo-controlled trial involving 602 treatment-naïve advanced HCC patients. ^8^ The median overall survival (OS) was significantly prolonged by 2.8 months (10.7 vs. 7.9 months, p<0.001) with sorafenib use, representing a 31% mortality risk reduction. The Asian-Pacific multicenter randomized controlled trial on sorafenib yielded similar results, yielding a median survival benefit of 2.3 months (6.5 vs 4.2 months, p=0.014). ^18^ The safety of sorafenib was further demonstrated in a multicenter prospective study (GIDEON study), supporting its use as a first-line therapy in advanced HCC. ^19^ As the first-in-class agent, sorafenib has initially been considered the gold standard for HCC systemic therapy. However, its exact mechanism of action remains poorly understood, and disease control rates of sorafenib in advanced HCC patients is below 50%. ^8^ Resistance to sorafenib can also develop through various mechanisms including epigenetic alterations, upregulation of drug efflux transporters and tumor microenvironment adaptation. ^20^ Common adverse reactions to sorafenib include hand-foot skin reaction, diarrhea, fatigue, and anorexia.

The encouraging results of sorafenib sparked hope in the search for additional systemic therapies for advanced HCC; however, in the decade following the publication of the SHARP trial, targeted therapy agents including sunitinib, brivanib, linifanib, and erlotinib all failed to meet the primary survival endpoints and were not superior to sorafenib. ^21^^–^^24^ Studies on second-line agents after failing sorafenib, such as tivantinib, have also remained fruitless. ^25^ This ultimately changed in 2018 when data on lenvatinib were published.

### Lenvatinib

Lenvatinib is another multiple TKI targeting epidermal growth factor receptor (EGFR), fibroblast growth factor receptor (FGFR), VEGFR, and PDGFR. ^26^ Its role in advanced HCC was first reported in a phase II trial in Japan and Korea, showing partial radiological response in 37% of patients and stable disease in 41% of patients after follow-up for over 2 years. ^27^ The landmark REFLECT trial involving head-to-head comparison between lenvatinib and sorafenib was published in 2018. Lenvatinib, when compared with sorafenib, demonstrated non-inferior median OS (13.6 vs 12.3 months) with significantly improved progression-free survival (PFS), median-time to progression, and objective response rate (ORR). ^28^ A recent meta-analysis reported that lenvatinib outperforms sorafenib in terms of OS, PFS, ORR and disease control, particularly in HBV-related HCC. ^29^ With support of the above data, lenvatinib monotherapy was first approved by the FDA in 2018, and has remained as a first-line systemic therapy option in HCC.

### Other targeted therapies

A range of other targeted therapies, particularly second-line therapy, have emerged after lenvatinib treatment. Regorafenib, a derivative of sorafenib with higher potency against VEGFR, was designed with higher anti-angiogenic activity and approved by the FDA in 2017. The RESOURCE trial compared regorafenib with placebo in patients who had failed sorafenib treatment, showing a significantly prolonged median OS by 2.8 months (10.6 vs 7.8 months, hazard ratio [HR] 0.63, p<0.001). ^30^ The survival benefit of regorafenib over placebo was further demonstrated in an extended analysis of the RESOURCE trial, supporting it as second-line therapy for patients who have failed sorafenib. ^31^

Cabozantinib is another multiple TKI with activity against VEGF, MET and AXL. In the CELESTIAL trial on patients who failed sorafenib, cabozantinib was shown to prolong median OS by 2.2 months (10.2 vs 8.0 months, p=0.005) and PFS by 3.3 months (HR for disease progression/death 0.44, p<0.001). ^32^ It has been postulated that the effect of cabozantinib is due to its additional activity against MET, the driver gene in sorafenib resistance. Due to the lack of data comparing regorafenib and cabozantinib against first-line TKIs, their role as first-line systemic therapies for HCC remains unanswered.

Donafenib, another sorafenib derivative with reduced hepatic metabolism, was tested against sorafenib in China, showing superior median OS (12.1 vs 10.3 months, p=0.0245) and higher 18-month survival (35.4% vs 28.1%, p=0.0460). ^33^ Apatinib, an oral VEGFR-2 inhibitor, has also been studied in a phase III trial in China and led to significant improvement in OS (8.7 vs 6.8 months, p=0.048). ^34^ Nonetheless, donafenib and apatinib are only available in China and have not been studied in non-Chinese populations.

Unlike the aforementioned small-molecule TKIs, ramucirumab is a recombinant immunoglobulin that inhibits angiogenesis via VEGFR-2 suppression. A phase II study evaluated ramucirumab monotherapy in treatment-naïve advanced HCC, with a median OS of 12 months, PFS of 4 months, and ORR of 9.5%. ^35^ The promising early results led to a phase III trial of ramucirumab in patients who failed sorafenib therapy (REACH trial). In the REACH trial, ramucirumab showed improvement in survival, albeit not reaching statistical significance. ^36^ The REACH-2 trial provided subgroup analysis, demonstrating that ramucirumab had higher efficacy in patients with alpha fetoprotein (AFP) level ≥400 ng/mL, and this group of patients had prolonged median OS by 1.2 months (8.5 vs 7.3 months, p=0.0199) and improvement in PFS (2.8 vs 1.6 months, p<0.0001). ^37^ Analysis on the expanded cohort for REACH-2 showed comparable results when the pre-requisite of AFP ≥400 ng/mL was added to patient recruitment. ^38^ These results highlight the heterogenous clinical characteristics of HCC, which can heavily influence the prognosis and choice of treatment. ^39^ Clear characterization of the etiology and biochemical profiles of HCC may be beneficial in optimizing treatment plans in advanced HCC.

As most trials have compared targeted therapy with placebo or sorafenib, there is no clear conclusion on the optimal second-line treatment. A systematic review suggested that regorafenib outperformed both cabozantinib and ramucirumab in patients with AFP <400ng/mL, while survival outcomes were similar for all three agents in patients with AFP ≥400 ng/mL. ^40^ More recent studies have pooled data from published trials to compare regorafenib and cabozantinib as salvage therapy after sorafenib failure. Kelley et al. ^41^ pooled data from the CELESTIAL and RESOURCE trials and showed that cabozantinib had comparable OS and PFS to regorafenib. In contrast, Merle et al. ^42^ suggested that regorafenib may trend towards better OS and have a more favorable side-effect profile when compared with cabozantinib. Head-to-head trials are necessary to determine optimal second-line targeted therapies for advanced HCC.

## Immunotherapy

### Therapeutic targets

Immune system evasion is a key pathophysiological feature of various cancers. The reversal of immune evasion has emerged as a key therapeutic target in oncology. ^43^ Immune checkpoints are cell surface receptors that suppress T-cell function, leading to immunotolerance. While immune checkpoints play critical roles in preventing autoimmunity, tumor cells exploit this mechanism to achieve immune evasion and avoid T-cell mediated tumor cytotoxicity. ^44^ HCC frequently occurs in chronically inflamed cirrhotic livers, which enrich intra-tumoral cancer-associated fibroblasts and tumor-associated macrophages. ^45^ These cells express high levels of immune checkpoints, deactivate tumor-specific CD8+ cytotoxic T cells, and promote immunosuppressive regulatory T-cell differentiation. ^46^ Based on these mechanisms, immune checkpoint inhibition has been hypothesized to be efficacious in HCC ( Figure 1).

Cytotoxic T-lymphocyte-associated antigen 4 (CTLA4), programmed cell death protein 1, and its ligand (PD1 and PDL1) are well-characterized actionable immune checkpoints. ^47^^,^^48^ In particular, the combination of PD1 and CTLA4 inhibition may have synergistic effects, as CTLA4 acts upstream of PD1 in the priming stage and has additional effects on regulatory T cells. ^49^ Hence, immune checkpoint inhibitors (ICIs) have been developed for the blockade of these checkpoints, and have demonstrated success in melanoma, lung cancer, and renal cell carcinoma. ^50^^–^^52^ Its use has expanded to HCC, with multiple agents demonstrating positive results in clinical trials.

### Anti-PD1 monotherapy

Nivolumab is a human IgG4 monoclonal antibody targeting PD1. The CheckMate-040 phase II dose escalation/expansion trial showed an objective response to nivolumab monotherapy in 20% of HCC patients, with a median duration of response of 9.9 months. ^53^ In the phase III CheckMate-459 trial, which compared nivolumab against sorafenib in Child-Pugh A patients with advanced HCC, the nivolumab arm trended towards better OS; however, this difference did not reach statistical significance (16.4 vs 14.7 months, p=0.075). ^54^ Nonetheless, the safety profile of nivolumab was more favorable than sorafenib, with >50% reduction in grade-3 or -4 treatment-emergent adverse events, making nivolumab monotherapy a potential therapeutic option when TKIs are contraindicated. However, accelerated approval for nivolumab monotherapy was withdrawn by the US FDA Oncologic Drug Advisory Committee in 2021, based on the statistically non-significant survival benefit of nivolumab over sorafenib in the CheckMate-459 trial.

Pembrolizumab, another humanized anti-PD1 antibody, was tested against placebo as a second-line treatment in patients with HCC who failed sorafenib treatment (KEYNOTE-240 trial). Pembrolizumab led to median OS of 13.9 months, which was numerically higher than the 10.6 months in placebo, although this did not reach statistical significance. ^55^ A similarly designed trial in Asia (KEYNOTE-394 trial comparing pembrolizumab vs placebo in sorafenib-failed patients) showed significantly improved median OS (14.6 vs 13.0 months, p=0.018) and PFS (HR=0.47, p=0.0032) with pembrolizumab. ^56^

### Combination regimens involving immunotherapy

A combination of anti-PD1 (durvalumab) and anti-CTLA4 (tremelimumab) was developed based on its potential synergistic effects. In the STRIDE trial (single-dose tremelimumab + regular interval durvalumab), combination therapy led to superior survival benefits compared with durvalumab monotherapy (median OS 18.7 months in combination therapy vs 13.6 months in durvalumab monotherapy). ^57^ The combination of durvalumab + tremelimumab was further studied in the landmark HIMALAYA phase III trial and was granted formal FDA approval in October 2022. ^58^ The median OS in durvalumab + tremelimumab sequential therapy was superior to sorafenib (16.56 vs 13.77 months), with a reduced mortality risk of 0.78 (p=0.0035). ^59^ In the same trial, durvalumab monotherapy was also shown to be non-inferior to sorafenib. Comparison between durvalumab + tremelimumab and durvalumab monotherapy arms confirmed the survival benefits of CTLA4 blockade in addition to anti-PD1 (3-year survival 30.7% in combination therapy vs. 24.7% in durvalumab monotherapy).

Another combination of anti-PD1 + anti-CTLA4 is the combination of nivolumab and ipilimumab. The CheckMate-040 trial studied nivolumab + ipilimumab in patients who progressed on sorafenib treatment. ^60^ Combination treatment with nivolumab and ipilimumab led to an ORR of 32%, with a complete response in 5% of patients. The ORR was at least four-fold higher for nivolumab + ipilimumab than for other existing therapies (ORR of approximately 4-7%). Hence, the combination of nivolumab and ipilimumab has been granted accelerated approval for HCC patients with prior sorafenib treatment. ^61^

Although dual immunotherapy has shown synergistic effects, it may also lead to additive immune-related adverse events. ^62^ These include skin rash, diarrhea, colitis, pneumonitis, hepatitis, and hyper/hypothyroidism.

Another approach to immunotherapy is to combine ICIs with targeted therapy, with the prime example being the combination of atezolizumab (anti-PDL1) + bevacizumab (anti-VEGFR monoclonal antibody). It has been hypothesized that VEGF potentiates PDL1 effects, ^63^ and simultaneously blocking both receptors may lead to synergistic effects. The IMbrave150 phase III trial examined the efficacy of atezolizumab + bevacizumab against sorafenib monotherapy. ^64^ In the primary analysis, OS at 12 months was significantly prolonged in the atezolizumab + bevacizumab group (HR for mortality 0.58, p<0.001), and a similar pattern was seen for PFS (6.8 months in combination arm vs 4.3 months in sorafenib arm, HR for disease progression or death 0.59, p<0.001). Extended follow-up for 1-year after the primary endpoint demonstrated that the atezolizumab + bevacizumab combination had a sustained antitumor effect with a stable safety profile. ^65^ Bleeding complications in bevacizumab are well-documented; hence, patients recruited to the IMbrave150 trial were screened and treated for esophageal varices prior to the start of systemic therapy. The addition of atezolizumab did not increase the risk of gastrointestinal bleeding compared to bevacizumab monotherapy. Based on the IMbrave 150 trial results, the FDA approved the combined use of atezolizumab + bevacizumab in 2020 given its improved OS and PFS compared to sorafenib. ^66^ It is noteworthy that the IMbrave150 trial recruited patients with high-risk features (e.g., macrovascular invasion of major portal veins, >50% liver involvement, or biliary tract invasion), which are usually excluded in other major trials. Nonetheless, the patient population in IMbrave150 still had a relatively preserved liver reserve (Child-Pugh class A), and the use of atezolizumab + bevacizumab in patients with severely impaired liver function remains unanswered.

With encouraging results from atezolizumab + bevacizumab, other trials on ICIs + targeted therapies have also been reported ( Table 1). The regimens of atezolizumab + cabozantinib, pembrolizumab + lenvatinib, sintilimab (anti-PD1) + bevacizumab, and camrelizumab (anti-PD1) + rivoceranib (TKI) have all shown significant improvements in PFS over TKIs, although their effects on OS have been inconsistent. ^67^^–^^70^

## Expanding indications of systemic therapies

### Neoadjuvant treatment

Although systemic therapies have classically been used for unresectable advanced HCC, their potential role in neoadjuvant treatment has also been explored. Neoadjuvant cemiplimab (anti-PD1) prior to hepatectomy has been studied in a phase II trial on 21 patients with resectable HCC, and 20% of patients had significant tumor necrosis in the resected tumor sample. ^71^ A similar trial on 30 patients with resectable HCC reported that neoadjuvant use of nivolumab or nivolumab + ipilimumab prior to surgical resection led to significant tumor necrosis in approximately 30% of patients. ^72^ While these two studies highlighted the potential anti-tumor effect of systemic therapy prior to surgery, their impact on recurrence risk and survival remains uncertain.

In addition to patients with resectable HCC, systemic therapy has been studied for downstaging patients with unresectable disease. In a pilot study of 10 patients with major vessel invasion, neoadjuvant therapy with anti-PD1 + TKI led to successful downstaging in 100% of patients, with 80% of patients eventually undergoing hepatectomy. Among patients who underwent surgery, the 1-year recurrence-free survival rate was 75%. ^73^ A similar observational study assessed the neoadjuvant use of anti-PD1 + TKI in 63 patients with unresectable HCC, where 15.9% of patients had successful downstaging and received curative resection at a median of 3.2 months after initiation of systemic therapy. In the patients who received surgery, 60% had complete pathological response on the resected tumor, and 80% remained disease free after 11.2 months of follow-up. ^74^ In a phase Ib trial, neoadjuvant cabozantinib + nivolumab was studied in 15 patients with unresectable disease, with 12 patients achieving successful curative resection and 5 patients achieving major pathologic response. ^75^ These studies highlight the enormous potential of neoadjuvant systemic therapy in downstaging HCC to expand the curative treatment window. Further large-scale trials on neoadjuvant strategies are warranted.

### Adjuvant treatment

Adjuvant systemic therapy after curative HCC treatment has been studied as a strategy for reducing recurrence. The phase III STORM trial on adjuvant sorafenib after resection/ablation showed negative results, ^76^ and subsequent studies on adjuvant sorafenib have shown mixed results. ^77^ Despite the disappointment with adjuvant sorafenib, newer systemic agents may have better efficacy. The IMBrave050 trial recruited 668 patients who underwent resection/ablation with a high risk of recurrence, aiming to compare outcomes between adjuvant atezolizumab + bevacizumab and active surveillance. After 12 months of treatment, adjuvant atezolizumab + bevacizumab led to significantly improved recurrence-free survival compared to active surveillance (HR 0.72, p=0.012), ^78^ representing the first trial to report the benefits of adjuvant immunotherapy in HCC. Multiple adjuvant trials including CA209-9DX (nivolumab monotherapy), ^79^ EMERALD-2 (durvalumab +/- bevacizumab) ^80^ and KEYNOTE-937 (pembrolizumab) ^81^ are ongoing, and the results have important implications for the adjuvant use of systemic therapies.

### Combination of systemic therapies with loco-regional therapy

The combination of systemic and loco-regional therapies has generated interest in the use of immunotherapy, as loco-regional therapies may prime intratumoral immune activity and enhance the effects of systemic agents. ^82^

The combination of anti-PD1 + SBRT is one of the best-studied strategies involving both systemic and locoregional therapies. A case series in 2019 reported 5 BCLC stage C HCC patients who received anti-PD1 + SBRT, demonstrating a 100% response rate with no disease progression after a median follow-up of 14.9 months. ^83^ In a subsequent cohort study by the same group, ICIs + SBRT was superior to TACE in terms of PFS (93.3% vs. 16.7%, p<0.001) and OS (93.8% vs. 31.3%, p<0.001) after 1 year in 16 patients with BCLC-C HCC. ^84^ The use of ICIs + SBRT has further progressed to a phase I trial in 2023, demonstrating that nivolumab + ipilimumab + SBRT was superior to nivolumab + SBRT in terms of PFS (11.6 vs 2.7 months, p<0.05) and OS (41.6 vs 4.7 months, p<0.05). ^85^ The START-FIT phase II trial modified the combination regimen to TACE + SBRT + avelumab (anti-PDL1). Among 33 patients with locally advanced HCC who received this combination regimen, an impressive 55% of subjects were successfully downstaged and became amenable to curative therapy, with 42% having a complete radiological response. ^86^

The combination of systemic therapy with TACE has also been explored. A retrospective study of camrelizumab + TACE showed an ORR of 35.3% and OS of 13.3 months, ^87^ although this strategy has not been studied in clinical trials. The combination of sorafenib + TACE and lenvatinib + TACE has been studied in phase III trials, and both combinations led to a significant improvement in PFS by >60% when compared with TKI monotherapy. ^88^^,^^89^ In 2022, interim trial results on the combination of lenvatinib + camrelizumab/sintilimab + TACE were reported. Notably, this triple combination led to successful downstaging and hepatectomy in 50% of patients, with patients achieving a 48-week OS of 96.4%.

## Discussion

Systemic HCC treatment has evolved rapidly. Numerous trials have established the efficacy and safety of targeted therapies and immunotherapies, dramatically expanding the treatment armamentarium for patients with advanced HCC. Despite promising results from clinical trials, real-world data, particularly for newer agents, are limited. Patients in clinical trials are generally highly selected, with good premorbid and limited comorbidities. In contrast, HCC patient subgroups such as patients with advanced cirrhosis, high bleeding risk, renal impairment or cardiometabolic diseases are limited. ^90^ Among the under-studied patient groups, patients with advanced cirrhosis or decompensated disease may be particularly problematic, as these patients form a substantial proportion of HCC patients commonly encountered in clinical practice. Encouragingly, data on systemic therapy in Child-Pugh B HCC patients are accumulating and have demonstrated the potential impact of cirrhosis severity on disease outcomes. ^91^^–^^93^ Extensive efforts are required to establish the efficacy and safety of systemic therapy in these important patient groups.

HCC is a heterogeneous disease with substantial differences in etiology and tumor biology, ^94^ which may in turn affect treatment response and prognosis. For example, in the landmark SHARP trial on sorafenib, the median OS was highest in HCV patients (6.6 months), followed by HBV patients (3.6 months) and patients with alcohol-related liver disease (2.3 months). ^95^ Differential treatment outcomes have also been reported for immunotherapy, in which patients with non-viral HCC were less responsive to anti-PD1, and non-alcoholic steatohepatitis HCC was associated with significantly poorer survival. ^96^ Further mechanistic studies are required to study the interaction between background liver disease and systemic therapy efficacy.

In addition to subtyping HCC according to disease etiology, molecular subtyping may also have a growing role in clinical management. Current international guidelines generally recommend image-based diagnosis of HCC in high-risk patients, and histological proof may not be necessary. ^9^^,^^97^ However, some clinicians have proposed the benefits of tissue diagnosis, primarily for molecular phenotyping and to identify druggable targets. ^98^ As the therapeutic options for advanced HCC grow, the one-size-fits-all approach will no longer be appropriate, and personalized treatment decisions based on clinical and molecular parameters will be necessary.

Traditionally, advanced HCC has restricted treatment options and is invariably associated with poor prognosis. With better characterization of oncogenic signaling pathways and tumor immunological regulation, the landscape of systemic therapy for HCC has dramatically shifted in the past decade. In addition to improving the prognosis of patients with advanced HCC, systemic therapies may also be expanded to neoadjuvant/adjuvant treatment. Numerous large trials are currently ongoing and we anticipate emerging evidence in the field of systemic HCC therapy.

## Author contributions

TKHW was involved in literature review, data interpretation, and drafting of the manuscript. RWHH, LYM, JF, and WKS were involved in the data interpretation and critical revision of the manuscript. MFY was involved in study conception, critical revision of the manuscript, and overall study supervision. All authors have read and approved the final version of the manuscript.

## Data Availability

No data was associated with this article.
