# Supplementary material for: Hepatocellular carcinoma: Advances in systemic therapies
Source: F1000Res. 2024 May 7;13:104. Originally published 2024 Feb 16. [Version 2] doi: 10.12688/f1000research.145493.2 (PMC11099512; doi:10.12688/f1000research.145493.2)
Supplement: Peer review history

# Reviewer report: Reviewer response for version 2

**DOI:** 10.5256/f1000research.165333.r274814

**Published:** 2024-05-17

**Authors:** Leonardo Stella

**Affiliations:** 1 Fondazione Policlinico Universitario Gemelli IRCCS, Rome, Rome, Italy

I'd like to thank the authors for improving their review, as demanded.

 I have no further comments on this article, the authors answered all my questions.

Is the review written in accessible language?

Yes

Are all factual statements correct and adequately supported by citations?

Yes

Are the conclusions drawn appropriate in the context of the current research literature?

Yes

Is the topic of the review discussed comprehensively in the context of the current literature?

Partly

Reviewer Expertise:

HCC, cirrhosis, liver fibrosis,

I confirm that I have read this submission and believe that I have an appropriate level of expertise to confirm that it is of an acceptable scientific standard.

# Reviewer report: Reviewer response for version 2

**DOI:** 10.5256/f1000research.165333.r274813

**Published:** 2024-05-16

**Authors:** Won-Mook Choi

**Affiliations:** 1 Department of Gastroenterology, Liver Center, Asan Medical Center, University of Ulsan College of Medicine, Seoul, South Korea

The authors addressed all my comments properly, and I have no further comments.

Is the review written in accessible language?

Yes

Are all factual statements correct and adequately supported by citations?

Yes

Are the conclusions drawn appropriate in the context of the current research literature?

Yes

Is the topic of the review discussed comprehensively in the context of the current literature?

Yes

Reviewer Expertise:

Hepatocellular carcinoma; Chronic hepatitis B; Liver fibrogenesis

I confirm that I have read this submission and believe that I have an appropriate level of expertise to confirm that it is of an acceptable scientific standard.

# Reviewer report: Reviewer response for version 2

**DOI:** 10.5256/f1000research.165333.r274812

**Published:** 2024-05-16

**Authors:** Shang-Chin Huang

**Affiliations:** 1 National Taiwan University Hospital Beihu Branch, Taipei City, Taipei City, Taiwan; 2 College of Medicine, National Taiwan University (Ringgold ID: 33561), Taipei City, Taipei City, Taiwan

Thanks for your responsive revision. I have no further comments to make.

Is the review written in accessible language?

Yes

Are all factual statements correct and adequately supported by citations?

Yes

Are the conclusions drawn appropriate in the context of the current research literature?

Yes

Is the topic of the review discussed comprehensively in the context of the current literature?

Yes

Reviewer Expertise:

Hepatology

I confirm that I have read this submission and believe that I have an appropriate level of expertise to confirm that it is of an acceptable scientific standard.

# Reviewer report: Reviewer response for version 1

**DOI:** 10.5256/f1000research.159449.r254024

**Published:** 2024-04-17

**Authors:** Leonardo Stella

**Affiliations:** 1 Fondazione Policlinico Universitario Gemelli IRCCS, Rome, Rome, Italy

I really appreciate the work Trevor Kwan-Hung Wu et al. made with this review, that summarize last evidence about systemic treatment in hepatocellular carcinoma (HCC) landscape.

 Particularly, the review provides a comprehensive overview of systemic treatments for advanced HCC, spanning from sorafenib's introduction to recent combination therapies like atezolizumab plus bevacizumab or durvalumab plus tremelimumab. It explores expanded indications beyond advanced stages, including combination with locoregional therapies and potential neoadjuvant/adjuvant use. Acknowledging the importance of real-world efficacy, it highlights the need for further research in specific patient subgroups. Lastly, the study recognizes the evolving nature of HCC treatment, emphasizing personalized approaches based on advancing understanding and accumulating data.  On the other side, the review lacks of specific real-world data on systemic treatment in diverse patient subgroups, and authors should underline how comparative analysis of efficacy and safety among different therapies is also insufficient, limiting insights for treatment selection. Additionally, there's a call for deeper exploration of liver disease etiology's influence on treatment outcomes and the necessity for further research to elucidate optimal therapy utilization and long-term effects.

 There aren't major concerns. Some minor points are:

 - Authors should add last evidence on phase I/II studies who are leading to upcoming phase III trial on double ICI + bevacizumab are lacking, so I suggest including recent studies about double ICI + anti-VEGF:

 *Tiragolumab + Atezolizumab/Bevacizumab (Finn R, et. al., 2023 [Ref1 ])

 *Relatlimab + Nivolumab/Bevacizumab (Sangro B, et. al., 2023 [Ref 2])

 - Authors should expand the paragraph about triple combination (locoregional + TKI + ICI), and to write down latest news about Chinese studies about HAIC + TKI + ICI.

Is the review written in accessible language?

Yes

Are all factual statements correct and adequately supported by citations?

Yes

Are the conclusions drawn appropriate in the context of the current research literature?

Yes

Is the topic of the review discussed comprehensively in the context of the current literature?

Partly

Reviewer Expertise:

HCC, cirrhosis, liver fibrosis,

I confirm that I have read this submission and believe that I have an appropriate level of expertise to confirm that it is of an acceptable scientific standard, however I have significant reservations, as outlined above.

## References

[1] : Results from the MORPHEUS-liver study: Phase Ib/II randomized evaluation of tiragolumab (tira) in combination with atezolizumab (atezo) and bevacizumab (bev) in patients with unresectable, locally advanced or metastatic hepatocellular carcinoma (uHCC). *Journal of Clinical Oncology* .2023;41(16_suppl) : 10.1200/JCO.2023.41.16_suppl.4010 10.1200/JCO.2023.41.16_suppl.4010

[2] : RELATIVITY-106: A phase 1/2 trial of nivolumab (NIVO) + relatlimab (RELA) in combination with bevacizumab (BEV) in first-line (1L) hepatocellular carcinoma (HCC). *Journal of Clinical Oncology* .2023;41(4_suppl) : 10.1200/JCO.2023.41.4_suppl.TPS636 10.1200/JCO.2023.41.4_suppl.TPS636

# Response

**Published:** 2024-04-19

**Authors:** Man-Fung Yuen

**Affiliations:** University of Hong Kong, Hong Kong

**On the other side, the review lacks of specific real-world data on systemic treatment in diverse patient subgroups, and authors should underline how comparative analysis of efficacy and safety among different therapies is also insufficient, limiting insights for treatment selection.**

 Response: Thank you for your comments. We agree that there is a lack of real-world data in specific patient subgroups, and there is a lack of head-to-head studies between systemic therapy options. We have added additional discussion points in our article, highlighting the importance of further real-world data in guiding our treatment decisions. (Discussion section: Paragraph 1)

**Additionally, there's a call for deeper exploration of liver disease etiology's influence on treatment outcomes and the necessity for further research to elucidate optimal therapy utilization and long-term effects.**

 Response: Thank you very much for your insightful comments. We agree that the role of liver disease etiology on treatment outcomes is a critical area of research. We have expanded our Discussion section to further discuss the role of disease etiology on HCC treatment outcomes, highlighting the need for mechanistic studies and real-life data in this area. (Discussion section: 2 ^nd^ paragraph)

**There aren't major concerns. Some minor points are:**

**- Authors should add last evidence on phase I/II studies who are leading to upcoming phase III trial on double ICI + bevacizumab are lacking, so I suggest including recent studies about double ICI + anti-VEGF:**

*Tiragolumab + Atezolizumab/Bevacizumab (Finn R, et. al., 2023 [Ref1 ])

*Relatlimab + Nivolumab/Bevacizumab (Sangro B, et. al., 2023 [Ref 2])

 Response: Thank you for your comments. We have now added additional description about these 2 trials. (Combination regimens involving immunotherapy: 6 ^th^ paragraph)

**- Authors should expand the paragraph about triple combination (locoregional + TKI + ICI), and to write down latest news about Chinese studies about HAIC + TKI + ICI.**

 Response: Thank you for your comments, we have now expanded the paragraph on locoregional therapy + systemic therapy, and also included description on the Chinese studies on HAIC. (Combination of systemic therapies with loco-regional therapy section: 6 ^th^ paragraph)

# Reviewer report: Reviewer response for version 1

**DOI:** 10.5256/f1000research.159449.r254015

**Published:** 2024-03-23

**Authors:** Shang-Chin Huang

**Affiliations:** 1 National Taiwan University Hospital Beihu Branch, Taipei City, Taipei City, Taiwan; 2 College of Medicine, National Taiwan University (Ringgold ID: 33561), Taipei City, Taipei City, Taiwan

In this review article, Wu et al. thoroughly summarized the current therapeutic regimens and recent advancements in systemic therapies against HCC. The content is well-organized and comprehensive. The tables and figure are adequate and informative. Since the manuscript is of significant scientific merit, I have only a few suggestions for your reference.

1. Since this is a review article, readers may expect some expert opinions from the authors, in addition to the current summary of published studies and discussion. A paragraph or several key points of experts’ opinions on clinical perspective and future investigation direction may help at the end of the article.
2. HCC is a heterogeneous cancer arising from different background environments and etiology, as mentioned by the authors. A table or figure summarizing the distinctions of each therapy (ex., anti-PD1 was less effective against NASH-HCC than other etiology, Lenvatinib seemed more effective than sorafenib among HBV-HCC, …) may help guide future investigations and clinical relevance.

Is the review written in accessible language?

Yes

Are all factual statements correct and adequately supported by citations?

Yes

Are the conclusions drawn appropriate in the context of the current research literature?

Yes

Is the topic of the review discussed comprehensively in the context of the current literature?

Yes

Reviewer Expertise:

Hepatology

I confirm that I have read this submission and believe that I have an appropriate level of expertise to confirm that it is of an acceptable scientific standard, however I have significant reservations, as outlined above.

# Response

**Published:** 2024-04-19

**Authors:** Man-Fung Yuen

**Affiliations:** University of Hong Kong, Hong Kong

**Comment #1 Since this is a review article, readers may expect some expert opinions from the authors, in addition to the current summary of published studies and discussion. A paragraph or several key points of experts’ opinions on clinical perspective and future investigation direction may help at the end of the article.**

 Response: Thank you for your comments. We have added an additional paragraph at the end to discuss our opinions and future perspectives. (Discussion section: Last paragraph)

**Comment #2 HCC is a heterogeneous cancer arising from different background environments and etiology, as mentioned by the authors. A table or figure summarising the distinctions of each therapy (ex., anti-PD1 was less effective against NASH-HCC than other etiology, Lenvatinib seemed more effective than sorafenib among HBV-HCC, …) may help guide future investigations and clinical relevance.**

 Response: Thank you for your insightful comments. We have expanded our Discussion section to further discuss the role of disease etiology on HCC treatment outcomes, highlighting the critical need for mechanistic studies and real-life data in this area. (Discussion section: 2 ^nd^ paragraph)

# Reviewer report: Reviewer response for version 1

**DOI:** 10.5256/f1000research.159449.r254012

**Published:** 2024-03-21

**Authors:** Won-Mook Choi

**Affiliations:** 1 Department of Gastroenterology, Liver Center, Asan Medical Center, University of Ulsan College of Medicine, Seoul, South Korea

This review article well summarizes recent advancements in systemic therapy, including real-world studies as well as pivotal trials, and the expansion of therapeutic indications such as combination therapy with locoregional therapy. It provides a comprehensive overview of recent developments in the field. I hope the authors may find some of my suggestions useful for improving the manuscript.

**Comment#1:** Please provide the table summarizing major trials on neoadjuvant, adjuvant, and combination regimens involving systemic therapies. It will provide a clear overview of the current status of expanding indications of systemic therapies.

**Comment #2:** There are real-world studies and meta-analyses comparing immunotherapy-based treatment with TKIs in both 1st line and 2nd line or beyond settings. Summarizing these studies would enhance the manuscript.

**Comment #3: **Please distinguish between the STAH trial, which evaluated the efficacy of TACE plus sorafenib and did not show an extension in OS, and the LAUNCH trial, which evaluated the efficacy of TACE plus lenvatinib and confirmed an extension in OS, compared with each TKI monotherapy.

**Comment #4: **Sorafenib, regorafenib, and lenvatinib also exhibit VEGF inhibition effects. Please modify the Figure 1 accordingly.

Is the review written in accessible language?

Yes

Are all factual statements correct and adequately supported by citations?

Yes

Are the conclusions drawn appropriate in the context of the current research literature?

Yes

Is the topic of the review discussed comprehensively in the context of the current literature?

Yes

Reviewer Expertise:

Hepatocellular carcinoma; Chronic hepatitis B; Liver fibrogenesis

I confirm that I have read this submission and believe that I have an appropriate level of expertise to confirm that it is of an acceptable scientific standard, however I have significant reservations, as outlined above.

# Response

**Published:** 2024-04-19

**Authors:** Man-Fung Yuen

**Affiliations:** University of Hong Kong, Hong Kong

**Comment#1: Please provide the table summarising major trials on neoadjuvant, adjuvant, and combination regimens involving systemic therapies. It will provide a clear overview of the current status of expanding indications of systemic therapies.**

 Response: Thank you for your helpful comment. We have added a table to provide description on the major trials on neoadjuvant, adjuvant and combination regimens. We agree that this will make it clearer for readers. (Table 3)

**Comment #2: There are real-world studies and meta-analyses comparing immunotherapy-based treatment with TKIs in both 1st line and 2nd line or beyond settings. Summarising these studies would enhance the manuscript.**

 Response: Thank you for your comments. We have added a description on meta-analysis data about immunotherapy + TKI in 1 ^st^ line/ 2 ^nd^ line settings. (Combination regimens involving immunotherapy section: last paragraph)

**Comment #3: Please distinguish between the STAH trial, which evaluated the efficacy of TACE plus sorafenib and did not show an extension in OS, and the LAUNCH trial, which evaluated the efficacy of TACE plus lenvatinib and confirmed an extension in OS, compared with each TKI monotherapy.**

 Response: Thank you for your comments. We have now specifically mentioned the comparison between the STAH trial and the LAUNCH trial in our section on combination therapy. (Combination of systemic therapies with loco-regional therapy section: 3 ^rd^ paragraph)

**Comment #4: Sorafenib, regorafenib, and lenvatinib also exhibit VEGF inhibition effects. Please modify the Figure 1 accordingly.**

 Response: Thank you for your comments. We have updated Figure 1 accordingly.
